# Perception of infection risk and attitudes toward infection prevention and control measures among health sciences students in Morocco: a cross-sectional study

**DOI:** 10.3389/ijph.2026.1609531

**Published:** 2026-07-22

**Authors:** Hajar El Alouani, Ousmane Traoré, Babacar Touré

**Affiliations:** 1 International Faculty of Dental Medicine, Health Sciences Research Center, College of Health Sciences, International University of Rabat, Rabat, Morocco; 2 Department of Clinical Microbiology and Hospital Hygiene, Clermont Auvergne - CHU Clermont-Ferrand, Clermont-Ferrand, France

**Keywords:** attitudes, healthcare-associated infections, infection prevention and control, patient safety, risk perception

## Abstract

**Objectives:**

To assess infection risk perception, attitudes toward Infection Prevention and Control (IPC) measures, and knowledge related to Healthcare-Associated Infections (HAIs) among health sciences students.

**Methods:**

A cross-sectional study was conducted among students enrolled in medical, dental, pharmacy, paramedical, and other health-related programs. Data were collected using a structured self-administered questionnaire. Statistical analyses included chi-square, Mann–Whitney U, and Kruskal–Wallis tests, as appropriate. Internal consistency was assessed using Cronbach’s alpha.

**Results:**

A total of 270 students were included in the study. Most participants had heard about HAIs (80.7%) and correctly identified their definition (94.4%). Surgical site infections were the most frequently identified HAIs (75.6%). The mean knowledge score was 4.32 ± 1.30, while attitudes toward IPC measures showed a high overall score (4.31 ± 0.55). The overall risk perception score was (3.01 ± 0.68), with university hospitals perceived as the highest-risk setting. Knowledge scores differed significantly according to field of study and prior IPC training (p < 0.05).

**Conclusion:**

Favorable attitudes were observed, but variations in knowledge and risk perception highlight the need to strengthen IPC training.

## Introduction

Healthcare-associated infections (HAIs) represent one of the major challenges to patient safety worldwide and continue to impose a substantial burden on healthcare systems. According to the World Health Organization (WHO), hundreds of millions of patients acquire an HAI each year. Incidence rates are up to three times higher in low- and middle-income countries (LMICs), where infection prevention and control (IPC) infrastructure and implementation remains heterogeneous and often insufficient [1]. HAIs are associated with a significant increase in morbidity, mortality, length of hospital stay, and healthcare costs, resulting in a substantial financial burden [2, 3]. In 2016, between 7.2 and 14.9 billion US dollars were spent on HAIs in the United States, thereby compromising overall quality of care and patient trust [4].

Infection prevention and control therefore constitutes a central pillar of patient safety and healthcare quality. Its effectiveness relies on the adherence of all healthcare personnel to standard precautions, including hand hygiene, appropriate use of personal protective equipment, waste management, compliance with additional precautions, and epidemiological surveillance [1]. Several studies have shown that the performance of IPC programs is closely linked to the level of knowledge, attitudes, and behaviors of healthcare professionals, as well as students and interns who represent the future hospital workforce [5, 6].

Students in the health sciences occupy a key position in the prevention of HAIs. From their first clinical placements, they are exposed to infectious risks and required to engage with various IPC practices [7]. Consequently, their preparedness regarding IPC measures may influence both their own safety and the quality of care delivered during clinical placements. Insufficient knowledge, limited awareness of infectious risks, or unfavorable attitudes toward preventive measures may contribute to inadequate implementation of IPC practices in healthcare settings [8].

In several countries, such as India [9], Peru [10], and Ethiopia [11], studies have also highlighted shortcomings among health sciences students, revealing significant gaps in knowledge of standard precautions, poor understanding of biological risks, and low adherence to preventive measures.

In Morocco, efforts to strengthen IPC programs have increased in recent years, particularly in university hospital settings. However, data regarding IPC-related preparedness among health sciences students remain limited. Most available studies have primarily focused on HAIs surveillance or hospital IPC program evaluation rather than students’ perception and attitudes toward IPC measures [12]. This lack of data limits the ability of universities and hospital institutions to implement targeted actions based on scientific evidence. In this context, the present study aims to assess perception of infection risk and attitudes toward IPC measures and knowledge related to HAIs among health sciences students in Morocco. The study also explored differences according to academic and institutional characteristics, including field of study, type of institution, prior IPC training, and clinical exposure as factors associated with these dimensions.

## Methods

### Type and study design

A descriptive cross-sectional study was conducted between July and October 2025 to assess infection risk perception, attitudes toward IPC measures, and knowledge related to HAIs among health sciences students.

### Study population and sampling

The study included students enrolled in various health-related training programs, including medicine, dentistry, pharmacy, paramedical sciences, and other health disciplines (medical biotechnology, health management, advanced practice, etc.).

A consecutive convenience sampling approach was used. All eligible students available during the study period who agreed to participate and complete the questionnaire were consecutively included. Students in initial training who had already completed or were planning to undertake a clinical internship were eligible for inclusion. Incomplete questionnaires were excluded from the analysis.

### Data collection tool

Data were collected using a self-administered questionnaire designed based on the international literature on infection prevention and control (IPC) and validated tools used in similar studies [13, 14]. The questionnaire consisted of four sections:

Sociodemographic data: age, sex, field of study, type of institution and IPC training received.

Knowledge of HAIs: covering the definition of HAIs, the most common infections, and preventive measures.

Attitudes toward IPC measures: Assessed using a 5-point Likert scale (1 = not effective, 2 = slightly effective, 3 = moderately effective, 4 = effective, and 5 = very effective).

Risk perception: Assessment of perceived risk in different care contexts using a 5-point Likert scale(1 = very low risk, 2 = low risk, 3 = moderate risk, 4 = high risk, and 5 = very high risk). The questionnaire was pretested with 20 students from different health-related programs to ensure the clarity and consistency of the items.

### Variables and scoring

The knowledge score was calculated as the sum of correct responses to dichotomous items, with scores ranging from 0 to 7. Higher scores indicated higher levels of knowledge related to HAIs.

The attitude score was obtained by calculating the mean of items assessed on a five-point Likert scale. Higher scores reflected more favorable attitudes toward IPC measures.

The risk perception score was calculated as the mean of responses to four items assessing the perceived level of risk in different healthcare settings.

### Statistical analyses

Statistical analyses were performed using Jamovi software, version 2.6.44. Qualitative variables were described using frequencies and percentages, whereas quantitative variables were presented as means and standard deviations. The internal consistency of the questionnaire scales was assessed using Cronbach’s alpha. Non-parametric tests were used for comparative analyses. Mann–Whitney U tests were applied for comparisons between two independent groups, such as sex, institution type, and prior IPC education. Kruskal–Wallis tests were used for comparisons involving more than two groups, particularly field of study. When statistically significant differences were identified, post-hoc pairwise comparisons were performed using the Dwass–Steel–Critchlow–Fligner method. Associations between qualitative variables were assessed using the χ^2^ test. Statistical significance was set at p < 0.05.

## Results

### Sociodemographic characteristics of the sample

A total of 270 students participated in this study, of whom 71.1% were female and 28.9% were male. The age of participants was mainly distributed into two groups: under 20 years (66.3%) and 20–35 years (33.7%).

Regarding field of study, students were primarily enrolled in medicine (36.7%), dentistry (28.5%), paramedical sciences (25.2%), and pharmacy (3.7%). The group of other health-related programs (5.9%) notably included students enrolled in medical biotechnology, health management, and master’s programs in advanced health practices.

Most participants were enrolled in semi-public or private institutions (71.7%), followed by public institutions (25.3%). A proportion of 2.6% did not report the type of institution attended.

Table 1 presents the sociodemographic characteristics of the participants.

**TABLE 1:** Sociodemographic characteristics of participants, Morocco, 2025 (N = 270).

Variables	Categories	N	%
Sex Age group Field of study Institution	Female Male <20 years 20–35 years Medicine Dentistry Pharmacy Paramedical Sciences/Care Health sciences (other programs) Public Semi-public/private Not reported	192 78 179 89 99 77 10 68 16 68 193 8	71.1 28.9 66.3 33.7 36.7 28.5 3.7 25.2 5.9 25.3 71.7 3.0

N= number of respondents.

% = Percentage.

### Students’ knowledge of HAIs

Overall, 80.7% of participants reported having previously heard about HAIs, and 94.4% correctly identified their definition. A statistically significant association was observed between prior IPC education and awareness of HAIs (χ^2^ = 42.7; p < 0.001). Among students who had received IPC-related training, 96.1% had previously heard about HAIs, compared with 62.3% among those without prior IPC education.

**FIGURE 1:**
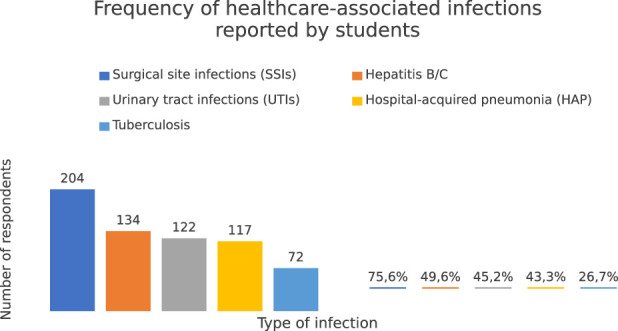
Distribution of the most frequently reported types of infections, Morocco, 2025 (N = 270) N = 270 respondents. Multiple responses were allowed; therefore, percentages do not sum to 100%.

Figure 1 details the types of infections perceived as most frequently associated with healthcare. The most commonly identified infections were surgical site infections (75.6%), followed by hepatitis B/C (49.6%), urinary tract infections (45.2%), and nosocomial pneumonia (43.3%). Mycobacterial infections were cited by 26.7% of respondents.

The overall knowledge level showed a mean score of (4.31 ± 1.27).

Comparative analysis did not demonstrate a statistically significant difference in knowledge according to sex (p = 0.594). In contrast, significant differences were observed according to the field of study (p < 0.001). Students enrolled in paramedical sciences achieved the highest mean score (4.85 ± 1.07), followed by students from other health-related programs (4.81 ± 1.22). Scores were lower in medicine (4.25 ± 1.29), dentistry (3.94 ± 1.20), and pharmacy (3.40 ± 1.58).

Analyses demonstrated significantly higher scores among paramedical science students compared with dentistry students (p < 0.001) and pharmacy students (p = 0.029).

A statistically significant variation was also observed according to the type of institution (p = 0.017), with higher scores among students enrolled in public institutions (4.71 ± 1.26) compared to those in semi-public/private institutions (4.16 ± 1.25). The category of students who did not declare their type of institution had a score of (4.75 ± 1.28). Similarly, students who received IPC-related education demonstrated significantly higher score (4.59 ± 1.17) than those who did not (4.01 ± 1.38) (p = 0.001).

In addition to the knowledge assessed, students were asked about their main sources of information regarding the prevention of healthcare-associated infections. University courses were the most frequently cited source (83.7%), followed by clinical internships (70.0%) and official documents (68.9%). Social media was used by 34.1% of participants, while 23.0% reported obtaining information from student associations. Conversely, other sources of information were very rarely used, involving only a negligible proportion of participants (0.4%).

Overall, knowledge levels varied across training programs and prior IPC education, highlighting potential differences in preparedness that may influence students’ adherence to preventive measures and perception of infection risk.

### Attitudes toward IPC measures

Students’ attitudes toward the perceived effectiveness of IPC measures are presented in Table 2. Response levels ranged from 1 (not effective) to 5 (very effective). Hand hygiene, glove use, mask use, isolation of infected patients, surface cleaning, and antibiotic prophylaxis were assessed using this scale.

**TABLE 2:** Perception of the effectiveness of Infection Prevention and Control measures, Morocco, 2025 (N = 270).

Measure	Not at all important (%)	Slightly important (%)	Moderately important (%)	Important (%)	Very important (%)
Hand hygiene Glove use Mask use Isolation Cleaning Antibiotic prophylaxis	1.1 1.9 0.7 1.9 0.4 1.1	9.3 3.7 2.6 1.9 1.1 5.6	18.1 12.6 14.1 4.4 0.7 16.7	34.1 33.3 34.8 23.0 17.4 38.1	37.4 48.5 47.8 68.9 80.4 38.5

% = Percentage.

Overall, students expressed a high level of positive attitudes, as reflected by the cumulative proportions of “effective” and “very effective” responses. The highest percentages concerned surface and equipment cleaning, considered effective by 97.8% of participants, followed by isolation of infected patients at 91.9%. Mask use 82.6% and glove use 81.8% were also widely perceived as effective. Hand hygiene was considered effective by 71.5% of participants, while antibiotic prophylaxis was considered effective by 76.6% of students.

The overall attitude score had a mean of (4.31 ± 0.53). The attitude scale showed acceptable internal consistency (Cronbach’s α = 0.685).

Comparative analyses show that attitudes toward prevention measures do not differ significantly by sex: women had a mean score of (4.34 ± 0.54), compared to (4.25 ± 0.52) for men (p = 0.158). No significant difference was observed according to the field of study (p = 0.139), with relatively homogeneous attitude levels among students in medicine, dentistry, pharmacy, paramedical sciences, and other health programs, all averaging around 4.2 to 4.4. Similarly, the type of institution was not associated with attitudes (p = 0.862): students in public and semi-public/private institutions had comparable mean scores, ranging from 4.28 to 4.35. IPC education also did not influence attitudes, with a score of (4.33 ± 0.54) among trained students and (4.31 ± 0.53) among untrained students (p = 0.675).

### Perception of infection risk

Perception of risk related to HAIs is presented in Table 3. The overall score had a mean of (3.00 ± 0.66), and values ranging from 1.00 to 4.50. The risk perception scale showed lower internal consistency (Cronbach’s α = 0.465), likely reflecting the heterogeneity of the healthcare settings assessed.

**TABLE 3:** Risk perception according to care settings, Morocco, 2025.

Care setting	Very low N %	Low N %	Moderate N %	High N %	Very high N %	Mean ± SD
University hospital	16 (5.9%)	42 (15.6%)	87 (32.2%)	72 (26.7%)	53 (19.6%)	3.39 ± 1.09
General practice office	44 (16.3%)	107 (39.6%)	79 (29.3%)	30 (11.1%)	10 (3.7%)	2.47 ± 0.98
Dental office	26 (9.6%)	79 (29.3%)	84 (31.1%)	50 (18.5%)	31 (11.5%)	2.94 ± 1.12
Home care	38 (14.1%)	57 (21.1%)	63 (23.3%)	49 (18.1%)	63 (23.3%)	3.15 ± 1.29

N= number of respondents.

% = Percentage.

Perceived risk levels varied according to the context: university hospitals were considered the highest risk (3.39 ± 1.09), with 32.2% of responses rated as moderate, 26.7% as high, and 19.6% as very high. Home care also presented a notable risk (3.15 ± 1.29), with 23.3% of responses very high and 18.1% high. Dental offices had a mean score of (2.94 ± 1.12), while general practice offices were perceived as the least risky (2.47 ± 0.98), with 55.9% of students assigning a very low to low level of risk. Overall, perceived infection risk was not uniform across healthcare settings, with higher scores observed in university hospitals and home care settings than in general medical practices.

Comparative analyses did not demonstrate statistically significant differences in perception scores according to sex (p = 0.105). Similarly, no significant difference was found according to the field of study (p = 0.179), with mean scores ranging from 2.9 to 3.2 across all programs, including medicine, dentistry, pharmacy, paramedical sciences, and other health disciplines. IPC education was not associated with perception levels: trained students had a mean of (3.02 ± 0.69), while untrained students had a nearly identical value (3.01 ± 0.66), (p = 0.849). In contrast, a significant difference was observed according to the type of institution (p = 0.043). Students enrolled in semi-public or private institutions had a mean score of (2.97 ± 0.66), whereas those in public institutions had a mean of (3.12 ± 0.65).

These findings highlight substantial variation in perceived infection risk across healthcare settings.

### Barriers to IPC implementation

The barriers reported by participants are presented in Table 4. The most frequently reported obstacles were non-compliance with protocols, insufficient equipment availability, lack of training, and excessive workload.

**TABLE 4:** Distribution of barriers to Infection Prevention and Control implementation, Morocco, 2025 (N = 270).

Barriers	N	%
Non-compliance with protocols Lack of equipment Lack of training Workload Other	184 177 160 139 1	68.1 65.6 59.3 51.5 0.4

N= number of respondents.

% = Percentage.

## Discussion

This study provides evidence on knowledge, attitudes, and risk perception related to infection prevention and control (IPC) among health sciences students and contributes to the growing body of literature examining IPC preparedness prior to entry into professional practice. By integrating multiple health disciplines, the findings offer a comprehensive overview of IPC related competencies at an early stage of clinical exposure.

In this study, the predominance of female participants in the sample aligns with the increasing feminization of health training programs reported in the literature. Studies conducted in India and Zambia have reported female proportions ranging from 57.2% to 73% among health sciences students [9, 13]. Similarly, the mean age of participants, centered around the twenties, is consistent with data from studies conducted in Africa and Asia [14, 15].

Regarding knowledge, 80.7% of participants had previously heard of HAIs, and 94.4% were able to correctly identify their definition, suggesting an overall satisfactory level of knowledge. However, score analysis shows that a significant proportion of students were at an intermediate level, while a minority reached a high level. This profile is similar to that reported among medical students in Sudan, where gaps were observed in hand hygiene, appropriate use of personal protective equipment, and contamination risk management [16].

Conversely, several studies conducted among pharmacy, medical and other health sciences students in Zambia, India, Saudi Arabia, and Malaysia reported higher levels of knowledge [13, 17–19]. In Zambia, Mudenda et al. showed that 86.9% of pharmacy students had a good level of knowledge, a result higher than that observed in the present study with a mean score of 3.4/7 (48.6%). These differences could be related to better curricular integration of IPC or the impact of WHO awareness campaigns during the COVID-19 pandemic [20].

The results observed among dentistry students show similarities with the literature. Studies conducted in Yemen and South Africa indicate that, despite good risk perception regarding aerosols and cross-contamination, knowledge concerning sterilization and standard precautions remains limited [21, 22]. The findings of the present study confirm that clinical exposure, in the absence of structured teaching, is insufficient to ensure adequate mastery of IPC principles.

Among paramedical programs, knowledge scores were higher than those observed in other fields. In a cross-sectional study of 301 nursing students in the Western Cape [23], 47.4% demonstrated a good overall level of IPC knowledge, indicating a strong baseline understanding of IPC concepts. Similarly, a study among 121 nursing students at a tertiary care hospital reported that 35.5% had an excellent level of knowledge of standard precautions, with an additional 64.5% exhibiting at least average knowledge [24]. Another cross-sectional survey of 102 Bachelor of Science in Nursing students in Hyderabad found that 73.5% were knowledgeable about standard precautions, 79.4% understood hand hygiene procedures, and 64% were aware of personal protective equipment use [25]. This level, comparable to that reported among nursing students, could be attributed to their early, frequent, and direct exposure to care activities, placing them on the front lines of infection risk and reinforcing their awareness of infection prevention and control measures.

Attitudes toward prevention measures were generally very favorable, with a mean score of (4.31 ± 0.53), regardless of sex, field of study, or type of institution. However, as observed in Sudan and Saudi Arabia, a gap persists between positive attitudes and actual practical proficiency [16, 18]. Similar findings have been reported among nursing students in South Africa, where attitudes may be influenced by institutional constraints and lack of resources [23].

These findings could be justified through behavioral theory especially the Health Belief Model [26]. According to Green et al., preventive health behavior is determined by various variables which include perceived susceptibility, perceived severity, perceived benefits, perceived barriers, cues to action, and self-efficacy. In the present study, the high attitude score suggests that students generally recognized the usefulness of IPC measures, However, the moderate level of perceived infection risk may partly reflect the heterogeneity of the healthcare settings assessed, ranging from critical and high-exposure environments, such as university hospitals, to less critical or routine care contexts, such as general medical practices. This is important because positive attitudes alone may not ensure consistent adherence to IPC practices if students perceive the risk of infection as low or not directly relevant to them.

Risk perception varied across healthcare settings, suggesting that students did not perceive infection risk uniformly across care contexts. University hospitals were identified as the highest-risk environments, consistent with WHO data showing a higher prevalence of HAIs in tertiary hospitals in low- and middle-income countries. Evidently, up to 15% of hospitalized patients in these countries acquire at least one healthcare-associated infection, compared to approximately 7% in high-income countries, with significant associated mortality [1]. In contrast, general medical practices were perceived as lower-risk environments. This may be due to the assumption made by the students that infections are a bigger problem in more advanced care settings. In reality, infections can occur in any environment.

From a risk perception perspective, individuals are more likely to adopt preventive behaviors when they perceive a threat as both likely and serious, and when they believe that recommended measures are effective and feasible [26]. This moderate risk perception may partly explain the potential gap between knowledge, favorable attitudes, and actual IPC adherence. IPC education should not only focus on technical knowledge but also strengthen students’ ability to identify infection risks in diverse care contexts, including outpatient care, dental offices, and home care.

Analyses show that few sociodemographic factors significantly influence KAP scores, with the notable exception of participation in structured IPC training, a determinant also highlighted in several studies [14, 27]. This reinforces the need for systematic and harmonized IPC teaching across health sciences curricula. Educational interventions should include theoretical teaching, simulation-based training, supervised clinical practice, and repeated assessment of IPC competencies.

Finally, the main barriers identified by students: non-compliance with protocols, lack of equipment, insufficient training, and workload align with international findings and support the relevance of educational strategies based on simulation and the strengthening of practical skills [28]. These barriers also underline that IPC adherence is not only an individual responsibility, but also depends on institutional conditions, availability of resources, workload management, and role modeling by clinical supervisors.

Some limitations should be acknowledged. The self-administered questionnaire may have introduced social desirability bias, particularly for attitude items, and actual IPC practices were not directly observed. Missing responses, small subgroup sizes, and the specific educational context may also limit the precision and generalizability of the findings. Future studies should include larger multicenter samples and objective assessments such as direct observation or simulation-based evaluation.

This study shows that health science students generally demonstrate favorable attitudes toward infection prevention and control measures. However, their perception of infection risk varied across care settings, suggesting that risk awareness may depend on the clinical context considered. Improved structure and harmonization of IPC educational content may contribute to enhancing knowledge, strengthening risk awareness, and supporting adherence to prevention measures among future healthcare professionals.
